# Commentary: Dietary Glutamic Acid, Obesity, and Depressive Symptoms in Patients With Schizophrenia

**DOI:** 10.3389/fpsyt.2021.725786

**Published:** 2021-10-14

**Authors:** Tauseef A. Khan, John L. Sievenpiper, John D. Fernstrom

**Affiliations:** ^1^Department of Nutritional Sciences, Temerty Faculty of Medicine, University of Toronto, Toronto, ON, Canada; ^2^Toronto 3D Knowledge Synthesis and Clinical Trials Unit, St. Michael's Hospital, Toronto, ON, Canada; ^3^Clinical Nutrition and Risk Factor Modification Centre, St. Michael's Hospital, Toronto, ON, Canada; ^4^Division of Endocrinology & Metabolism, Department of Medicine, St. Michael's Hospital, Toronto, ON, Canada; ^5^Scientist, Li Ka Shing Knowledge Institute, St. Michael's Hospital, Toronto, ON, Canada; ^6^Department of Psychiatry, University of Pittsburgh School of Medicine, Pittsburgh, PA, United States; ^7^Department of Pharmacology & Chemical Biology, University of Pittsburgh School of Medicine, Pittsburgh, PA, United States

**Keywords:** diet, dietary protein, glutamic acid, MSG, obesity, schizophrenia, depression

## Introduction

Kumar et al. (1) collected self-reported 24-h recalls of foods and beverages consumed by patients with schizophrenia. From these data, they estimated 24-h intakes of dietary protein and the glutamic acid present in dietary protein. These glutamate intake estimates were evaluated to assess the relationship between 24-hr glutamate intake and depression [Beck Depression Inventory scale (BDI)]. They concluded that no significant relationship was found between dietary glutamate intake and mood. However, among those who were not obese, higher intakes of glutamate (i.e., protein) were associated with greater depression.

In the introduction, the investigators note that it is important to understand the importance of environmental factors in the development of psychiatric disorders. Their contribution concerns the dietary intake of glutamate, an amino acid which is also a brain neurotransmitter. Their interpretation of the findings in non-obese individuals seems to be that ingesting dietary protein, which contains glutamate, raises blood glutamate concentrations sufficiently to cause an increase in glutamate penetration into brain, where it acts on neuronal glutamate receptors to cause depression.

## Statistical Issues

The authors conclusions are based on a linear regression analysis of estimated dietary glutamic acid intake with BDI score. We are concerned about these results based upon non-normality of the data. In Table 1 of their paper, the BDI data appear to be non-normally distributed based upon mean (14.5 ± SD 10.2) and range (2–50). For any normally distributed variable, about two-thirds of the cases are within 1-SD of the mean (in this case, 4.3 and 24.7) and 95% will be within two SDs of the mean (in this case, −5.9 and 34.9) (2). In addition, the mean is smaller than twice the SD, which also indicates that the data are not normal (3). The data are clearly positively skewed. This indicates that most data points cluster at low ends of the BDI scale with outliers at the high end. This issue is exacerbated by the stratification by obesity, as it limits the number of observations in each group and the obese have the tendency for higher intake of glutamate (*p* = 0.086).

The linear regression analysis employed in this study assumes that the data have normal distribution (though strictly it should be the residuals which are assumed to be normal); without transformation of the BDI (e.g., log transformation to normalize the distribution) this approach becomes inappropriate (4, 5). This is made clear when we attempt to estimate BDI using their regression equation for the obesity group with reasonable assumptions. The resulting estimates are uninterpretable at best and most likely biased. The slope of BDI risk with glutamic acid intake (2.39 BDI per glutamic acid grams/day) appears exaggerated as the estimates include both negative and very high BDI values. Furthermore, it is possible that a linear or straight line itself might not be the best fit. A non-linear or threshold analysis may show interesting or even opposite results.

We believe that the conclusions based upon a linearity assumption in this paper are spurious. If the authors were to run the analysis with appropriate transformation or explore non-linear or threshold analysis using appropriate methods (6), then we surmise that the correction of the large skew would effectively flatten the slope and would make the relationship non-significant or clinically trivial.

We also note that the antipsychotics were not identified, but described as chlorpromazine therapeutic equivalents. Each antipsychotic (and antidepressant) should have been identified, and included in the analysis, as several have notable effects on body weight (7, 8). Moreover, smoking should have been included as a factor in this study. Many psychotic and depressed patients smoke (9), and smoking is associated with a lower body weight, while smoking cessation can increase body weight (10, 11).

## Dietary Glutamate and Brain Glutamate Neurotransmission: Metabolic Considerations

The authors infer in their introduction that ingesting glutamate can lead directly to increases in brain glutamate levels and adversely modify brain functions via its neurotransmitter role. However, in this case, they misunderstand key metabolic features of dietary glutamate handling by the body. Almost all glutamate ingested each day derives from dietary protein [glutamate is abundant in dietary proteins (12)]. Free glutamate [naturally present in foods and added as monosodium glutamate (MSG)] makes a modest contribution by comparison (12). During digestion and absorption, almost all glutamate present in food (95+%) is catabolized by enterocytes (13). Consequently, only a very small fraction of ingested glutamate enters the circulation. This explains why ingesting a protein-containing (and MSG-containing) meal raises plasma glutamate levels by no more than 2-fold (14, 15). Such increases in plasma glutamate are insufficient to push glutamate into brain, owing to the metabolic properties of capillary endothelial cells in brain. These cells are joined by tight junctions, and form the “blood-brain barrier” (BBB) (16). For glutamate, they employ several energy-dependent transporters that prevent glutamate passage from blood into brain (16). The result is that consuming large amounts of glutamate in food, even when it raises plasma glutamate, does not elevate brain glutamate levels. The articles Kumar et al. (1) cite to support glutamate administration affecting brain function involve giving direct systemic injections to animals, thus bypassing gastrointestinal metabolism (17–22). Such injections produce abnormally large increments in plasma glutamate, sufficient to overwhelm the BBB barrier to glutamate [17-fold increments or more are required (23)]. In humans, plasma glutamate concentrations can be raised as much as 10-fold under artificial conditions (a single, enormous, oral dose of MSG ingested in a liquid in the fasting state) (24, 25). However, even with such non-physiologic increases [humans do not willingly consume such large amounts of pure MSG, because it tastes unpleasant (26)], glutamate does not penetrate into brain or alter brain function (24).

A more likely path through which dietary glutamate could influence the brain is via its interaction in the alimentary canal with glutamate receptors that occur in the mouth, stomach and intestines. This route is physiologic. For example, glutamate in food binds to the amino acid taste receptor in the mouth [which in humans is tuned specifically to sodium glutamate (27)], leading ultimately to the perception of umami taste by the brain via the activation of sensory nerves in the face (28). The taste of sodium glutamate is sensed as pleasant, and is hypothesized to direct amino acid and protein-seeking behavior (28). Glutamate also binds to glutamate receptors on gastrointestinal vagal afferents that provide sensory information to brainstem and higher brain centers involved in the reflex control of GI and other functions (29). Behavioral effects, where observed in animals after consuming orally-administered glutamate [e.g., (30)], may thus be mediated via this indirect neural pathway, rather than by a direct chemical action of dietary glutamate in the brain.

Finally, Kumar et al. (1) have examined protein ingestion, not MSG ingestion. From a nutritional perspective, it would be unfortunate if those reading their article concluded that individuals with schizophrenia should reduce their daily intake of protein, particularly when many already appear to be consuming too little (Table 1). Current dietary guidelines set an adequate protein intake level at 0.8 g/kg/day (31).

## Author Contributions

All authors listed have made a substantial, direct and intellectual contribution to the work, and approved it for publication.

## Conflict of Interest

JS has received research support from the Canadian Foundation for Innovation, Ontario Research Fund, Province of Ontario Ministry of Research and Innovation and Science, Canadian Institutes of health Research (CIHR), Diabetes Canada, PSI Foundation, Banting and Best Diabetes Centre (BBDC), American Society for Nutrition (ASN), INC International Nut and Dried Fruit Council Foundation, National Dried Fruit Trade Association, National Honey Board (the U.S. Department of Agriculture [USDA] honey “Checkoff” program), International Life Sciences Institute (ILSI), Pulse Canada, Quaker Oats Center of Excellence, The United Soybean Board (the USDA soy “Checkoff” program), The Tate and Lyle Nutritional Research Fund at the University of Toronto, The Glycemic Control and Cardiovascular Disease in Type 2 Diabetes Fund at the University of Toronto (a fund established by the Alberta Pulse Growers), and The Nutrition Trialists Fund at the University of Toronto (a fund established by an inaugural donation from the Calorie Control Council). He has received in-kind food donations to support a randomized controlled trial from the Almond Board of California, California Walnut Commission, Peanut Institute, Barilla, Unilever/Upfield, Unico/Primo, Loblaw Companies, Quaker, Kellogg Canada, WhiteWave Foods/Danone, and Nutrartis. He has received travel support, speaker fees and/or honoraria from Diabetes Canada, Dairy Farmers of Canada, FoodMinds LLC, International Sweeteners Association, Nestlé, Pulse Canada, Canadian Society for Endocrinology and Metabolism (CSEM), GI Foundation, Abbott, General Mills, Biofortis, ASN, Northern Ontario School of Medicine, INC Nutrition Research & Education Foundation, European Food Safety Authority (EFSA), Comité Européen des Fabricants de Sucre (CEFS), Nutrition Communications, International Food Information Council (IFIC), Calorie Control Council, International Glutamate Technical Committee, and Physicians Committee for Responsible Medicine. He has or has had ad hoc consulting arrangements with Perkins Coie LLP, Tate & Lyle, Wirtschaftliche Vereinigung Zucker e.V., Danone, and Inquis Clinical Research. He is a member of the European Fruit Juice Association Scientific Expert Panel and former member of the Soy Nutrition Institute (SNI) Scientific Advisory Committee. He is on the Clinical Practice Guidelines Expert Committees of Diabetes Canada, European Association for the study of Diabetes (EASD), Canadian Cardiovascular Society (CCS), and Obesity Canada/Canadian Association of Bariatric Physicians and Surgeons. He serves or has served as an unpaid scientific advisor for the Food, Nutrition, and Safety Program (FNSP) and the Technical Committee on Carbohydrates of ILSI North America. He is a member of the International Carbohydrate Quality Consortium (ICQC), Executive Board Member of the Diabetes and Nutrition Study Group (DNSG) of the EASD, and Director of the Toronto 3D Knowledge Synthesis and Clinical Trials foundation. His wife is an employee of AB InBev. JF is a scientific adviser to the International Glutamate Technical Committee, Brussels, Belgium. The remaining authors declare that the research was conducted in the absence of any commercial or financial relationships that could be construed as a potential conflict of interest.

## Publisher's Note

All claims expressed in this article are solely those of the authors and do not necessarily represent those of their affiliated organizations, or those of the publisher, the editors and the reviewers. Any product that may be evaluated in this article, or claim that may be made by its manufacturer, is not guaranteed or endorsed by the publisher.
